# The complete mitochondrial genome of *Downesia tarsata* (Coleoptera: Chrysomelidae: Cassidinae)

**DOI:** 10.1080/23802359.2021.1899862

**Published:** 2021-03-19

**Authors:** Shengdi Zhang, Qingyun Guo, Jiasheng Xu, Xuexiong Wang, Xiaohua Dai

**Affiliations:** a Leafminer Group, School of Life Sciences, Gannan Normal University, Ganzhou, China; b National Navel-Orange Engineering Research Center, Ganzhou, China

**Keywords:** *Downesia tarsata*, Complete mitochondrial genome, phylogenomic analysis

## Abstract

*Downesia* species are leaf-mining beetles mainly feed on Poaceae plants in the tropical and subtropical areas in Asia. In this study, we firstly sequenced and reported the complete mitochondrial genome for the genus. The complete mitogenome of *Downesia tarsata* is 18,557 bp in length, including 13 protein-coding genes (PCG), 22 transfer RNA (tRNA), two ribosomal RNA (rRNA), and one AT-rich region. Phylogenomic analysis indicated that *D. tarsata* is closely related to *Agonita chinensis*, and the two species belong to the same tribe of Gonophorini. The complete mitochondrial genome of *D. tarsata* could help clarify the phylogenetic relationship among Cassidinae species.

*Downesia* species (Chrysomelidae: Coleoptera: Cassidinae) are leaf-mining beetles, their host plants are mainly Poaceae, and they distribute mainly in the tropical and subtropical areas in Asia (Staines 2015). *Downesia tarsata* was only found in Guangdong and Hong Kong in China (Staines 2015), but we also recorded it on *Miscanthus floridulus* (Poaceae) in Jiangxi. However, no genome information of 41 *Downesia* species has not been reported at present. In this study, the complete mitochondrial genome of *D. tarsata* was sequenced and annotated, which may help understand its evolutionary history and population genetics.

*Downesia tarsata* samples were collected from Doushuihu, Jiangxi Province, China (114.430E, 25.809 N). The catalog number is 2016XI29002 and 20161120905 for the insects and their host plants, respectively. Voucher specimens are deposited in Nanling Herbarium, Gannan Normal University (GNNU). The sampling adults were all stored in 100% ethanol at −80 °C. The whole genomic DNA was obtained from a single specimen’s head tissue following the manufacturer’s protocol of the TIA Namp Genomic DNA kit (TianGen, China). DNA was preserved at −20 °C and sent to Shanghai Personal Biotechnology Co., Ltd. for mitochondrial genome sequencing.

The circular mitogenome (GenBank accession No. MW176089) of *D. tarsata* was obtained by the next-generation sequencing (NGS) with the whole-genome shotgun (WGS) strategy on the Illumina MiSeq platform. It is 18,557 bp in length containing A 42.45%, C 12.47%, G 8.90%, and T 36.18% respectively. It has 13 protein-coding genes (PCG), 22 transfer RNA (tRNA), two ribosomal RNA (rRNA), and an AT-rich region. Three kinds of start codons (ATT, ATA, and ATG) are found in 13 PCGs; six genes end with TAA, two genes end with TAG, and the other genes have an incomplete stop codon, three are TA– and two are T––. The lengths of 22 tRNA genes range from 58 bp to 70 bp, having clover-leaf structures except for trnSer1, which lost the DHU arm. The rRNA genes, rrnS, rrnL, were found between trnSer1 and the control region. The 727-bp rrnS has an A + T content of 78.68%, while the 1195-bp rrnL length has an A + T content of 79.33%. Among the 37 genes, 23 genes (9 PCGs and 14 tRNAs) are located on the major strand (N-strand) and 14 genes (4 PCGs, 8 tRNAs, and 2 rRNAs) on the minor strand (J-strand) just as in other Cassidinae species (Guo et al. 2017).

The other four partial genomes and seven complete genomes of Chrysomeloidea species from Genbank are downloaded and aligned with the MAFFT program (Katoh and Standley, 2013). A maximum likelihood (ML) tree (Guindon and Gascuel, 2003) with 10,000 bootstrap replicates were performed using IQ-tree (Nguyen et al., 2015) and viewed by Figtree v1.4.3 (Rambaut, 2016). The ML phylogenetic tree indicated that *D. tarsata* is closely related to *Agonita chinensis* (Figure 1), and the two species belong to the same tribe of Gonophorini. The mitochondrial genome of *D. tarsata* could provide useful genetic information for further study on the evolutionary relationship of Cassidinae species.

**Figure 1.**
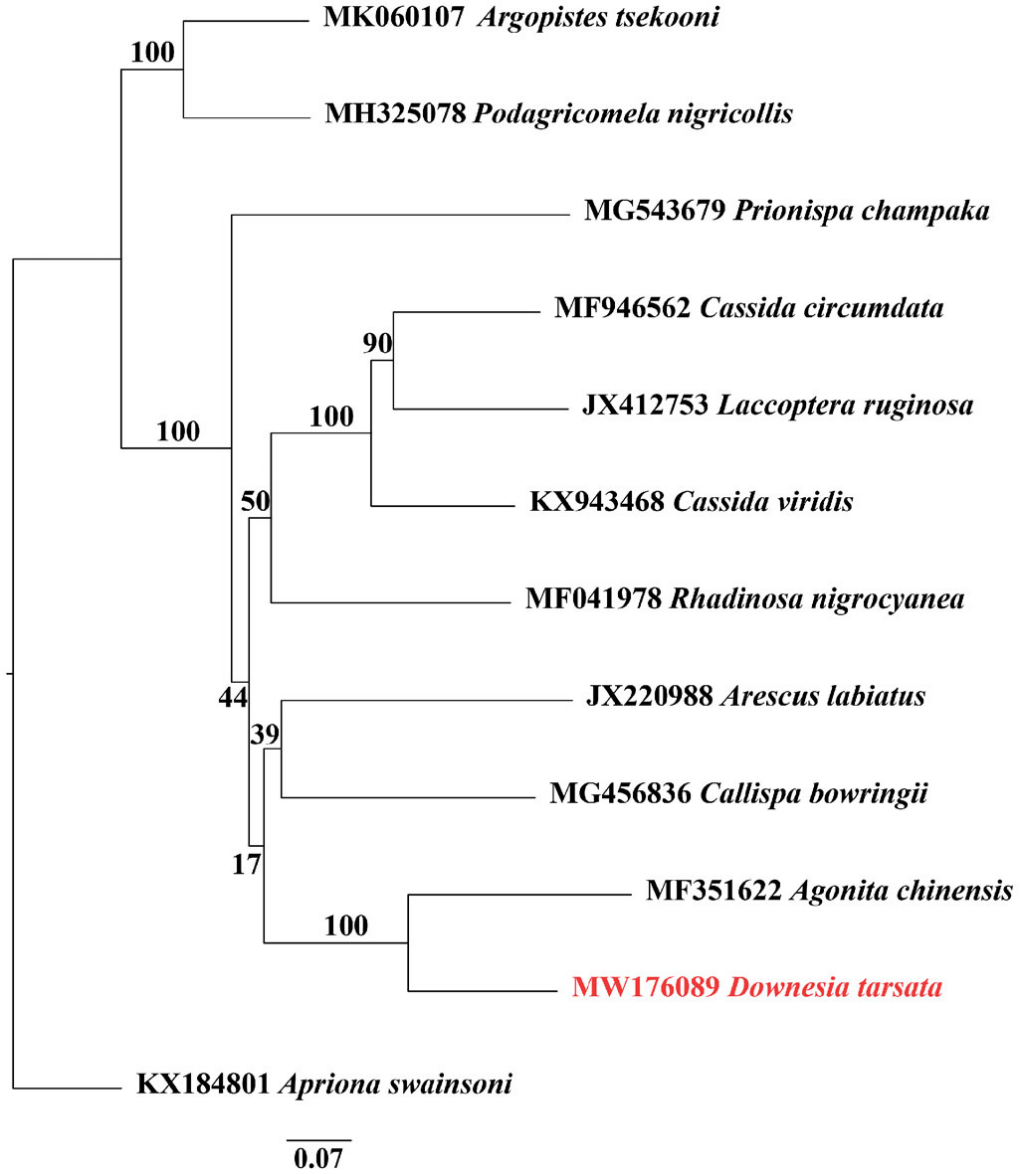
The maximum likelihood (ML) phylogenetic tree based on 12 mitochondrial genomes of Chrysomeloidea species. Branch values are ML bootstrap percentages.

## Data Availability

The genome sequence data that support the findings of this study are openly available in GenBank of NCBI at (https://www.ncbi.nlm.nih.gov/) under the accession no. MW176089. The associated **BioProject**, **SRA**, and **Bio-Sample** numbers are PRJNA678845, SRR13089188, and SAMN16815312 respectively.
